# Ready, willing, and able: understanding opioid overdose preparedness among college students in the southwestern United States

**DOI:** 10.3389/fpubh.2026.1837454

**Published:** 2026-05-05

**Authors:** Jake Samora, Kayla Lovett, Katie McCormick, Riya Shah, Sachi Kulkarni, Kasey Claborn

**Affiliations:** 1 School of Social Work, Addiction Research Institute, The University of Texas at Austin, Austin, TX, United States; 2 Department of Psychiatry and Behavioral Sciences, Dell Medical School, The University of Texas at Austin, Austin, TX, United States

**Keywords:** college students, emergency preparedness, overdose, prevention, young adults

## Abstract

**Introduction:**

The Ready, Willing, Able framework aids in examining emergency response preparedness among college campuses and student communities. There remains a gap in understanding levels of readiness, willingness, and ability in opioid overdose response among college students in the southwestern United States.

**Methods:**

We conducted a multiple methods protocol using a concurrent triangulation design. A cross-sectional survey was conducted among *N* = 352 college students at a university in the southwestern United States to measure readiness (i.e., knowledge of campus-based resources), willingness (i.e., comfort in spreading awareness of campus-based naloxone resources), and ability (i.e., reported knowledge of how to use naloxone). Descriptive analyses characterized levels of readiness, willingness, and ability among college students. Five focus groups were also conducted with *N* = 32 students who reported proximal experiences to overdose (i.e., personally experiencing, witnessing, or knowing someone who had experienced overdose) to understand factors that influenced readiness, willingness, and ability for overdose response preparedness, as well as perceived solutions to improve these domains among college students at their university. Applied thematic analysis and the Ready, Willing, Able framework guided qualitative analyses.

**Results:**

Survey findings characterized low levels of readiness, moderate levels of willingness, and low levels of ability for opioid overdose response preparedness. Emergent themes from applied thematic analysis revealed facilitating factors (e.g., accessing campus-based naloxone/training from various sources, targeting coursework into health-related tracks, granular details on overdose response protocol) and barriers (e.g., lack of advertisement of university resources, apathy/stigma toward overdose response preparedness, ineffective training) to overdose response preparedness. Students offered a multitude of solutions to improve readiness (e.g., expanding access to and awareness for university-based resources), willingness (e.g., empathy-based messaging to promote awareness), and ability (e.g., hands-on and step-by-step trainings) in overdose response preparedness.

**Conclusion:**

Findings highlighted areas of need among college students in improving opioid overdose response readiness, willingness, and ability in emergency preparedness. Student perspectives and expertise, as well as institutional resources (i.e., overdose education and naloxone distribution services, media-based awareness initiatives), should guide efforts to improve opioid overdose response preparedness on college campuses.

## 1 Introduction

From 1999 to 2018, the rate of opioid-only and polysubstance-involved overdose deaths among youth and young adults (i.e., individuals aged 13 to 25 years old) increased 384% and 760%, respectively (1). Between 2019 to 2021, median monthly overdose death rates among adolescent and young adult populations increased 109% and deaths involving fentanyl increased by 182% (2). College students report lifetime opioid misuse and prescription stimulant as high as 13% and 14%, respectively (3, 4), which highlights a risk profile for fatal overdose considering recent trends in drug-related morality involving psychostimulants and opioids (73% of stimulant-involved deaths from 2021 to 2024 involved opioids) (5). Specific risk factors for overdose among college-aged populations are indicated in polysubstance use with opioids and stimulants, structural vulnerability (i.e., unstable housing, parents with substance use problems), and mental health factors (6–8). Understanding college students' knowledge and awareness for overdose response preparedness is critical for improving response efforts on college campuses.

The Ready, Willing, Able (RWA) framework for examining public health emergency response provides a lens for viewing multidimensional aspects of preparedness for responding to opioid overdose among college students (9). Within the RWA framework, readiness is defined as systemic infrastructure, availability of tools, and strategic plans for responding to an emergency (9). Willingness is defined as the state of being inclined in mind or affect toward specific responses when a task is required by circumstances (9). Ability refers to the skill or knowledge an individual possesses to perform a task if permitted by the circumstances (9). Research has noted that applications of RWA offer potential for aiding assessment of organizational and individual-level preparedness among case examples of emergency responses supported by the Centers for Disease Control and Prevention (10). The RWA framework applied to understand deficits in emergency medical services preparedness during COVID-19 demonstrated that assessment and training informed by the RWA framework improved preparedness knowledge and interprofessional collaboration (11). In the context of overdose preparedness among college students, readiness is considered as awareness and participation in campus-based resources (i.e., naloxone training, distribution) (12). Willingness includes level of comfort seeking and sharing awareness of overdose response resources and preparedness (13). Factors influencing ability include confidence and knowledge of how to administer naloxone in the event of an opioid overdose (14).

College students report low levels of readiness and ability for responding to drug-related emergencies and overdose. Less than half of college students at a northeastern university possessed opioid-specific knowledge (i.e., knowledge of fentanyl as an opioid) and knowledge of opioid overdose symptomatology (15). A study conducted at a southeastern university found that only 16% of students had received training in naloxone administration (14). Campus-based naloxone distribution and training programs are beneficial for improving knowledge and attitudes in responding to opioid overdose (12). Peer education models for promoting awareness and knowledge of drug-related harms on college campuses are implemented at multiple university settings and show promise in increasing preparedness for drug-related emergencies and overdose (16, 17). Campus-based amnesty or “Good Samaritan” policies have been established at multiple universities to promote comfort in seeking emergency medical services when a college students experiences or witnesses a drug-related emergency (18, 19). Nevertheless, barriers to implementing campus-based overdose preparedness initiatives, including institutional and student-level stigma toward harm reduction programming, fear of punitive consequences for seeking emergency services, and perceptions of not being able to assist in drug-related emergencies (13, 20, 21) highlight the need to examine student preparedness to respond to opioid overdoses.

There remains a gap in examining (1) college student preparedness for opioid overdose response across RWA dimensions and (2) perceptions regarding preparedness from students with proximal experiences to drug overdose (i.e., students that have experienced overdose, witnessed overdose, or known someone who has experienced drug overdose). Further, there is a lack of research examining overdose and drug-related emergency preparedness among college students in the southwestern region of the United States, with overdose prevention research situated in this region noting social and legal barriers to community preparedness (22). This study employed multiple methods (i.e., cross-sectional survey, qualitative focus groups) to understand college students' knowledge about opioid overdose and response, perspectives on existing campus resources, and suggestions for improvement using the RWA framework for public health emergency response.

## 2 Materials and methods

We conducted a multiple methods study with a concurrent triangulation design (23) to understand students' readiness, willingness, and ability for opioid overdose response preparedness in the Spring of 2023. First, we surveyed students enrolled at a large university in the southwest region of the United States. The survey assessed awareness of and access to campus-based resources for opioid overdose, comfort levels discussing drug overdose and related resources, and knowledge of drug overdose prevention and response. We then conducted focus groups with students who had proximal experiences to overdose to provide a deeper understanding of what factors influenced relative levels of readiness, willingness, and ability to respond to opioid overdose. The University of Texas at Austin Institutional Review Board reviewed and approved all research activities.

### 2.1 Cross-sectional survey

#### 2.1.1 Procedure

Survey participants were recruited via convenience sampling through tabling outside the campus library weekly for a semester (16 weeks). Prospective student participants heard about the survey opportunity via passing by the table during a survey tabling event, social media, and word of mouth. Criteria for participation included (1) being 18 years of age or older, (2) ability to speak, read, and respond to survey items in English, and (3) current enrollment as an undergraduate or graduate student at the university. Study staff determined eligibility by asking questions related to the potential participant's age and verifying their student status via the student directory. Data were collected via REDCap, an online survey distribution platform which allowed participants to complete the study on their cell phone. Study procedures were conducted as follows: (1) interested participants approached the table, (2) participants were verified as age-eligible and enrolled students, (3) participants provided informed consent, (4) participants completed the online survey, (5) participants were thanked and compensated for their time. Participants who completed the survey were compensated with a $10 Amazon e-gift card. On average, participants completed the survey in 5–10 min.

#### 2.1.2 Measures and analysis

Demographics were collected as part of the survey, including age, race, ethnicity, and gender identity. Additionally, the year of coursework (i.e., freshman, sophomore, junior, senior, graduate, doctoral) and affiliations with campus-based organizations were recorded. Grounded in the RWA framework (9), we developed survey items measuring participant's awareness and engagement with overdose response infrastructure, such as naloxone training (i.e., readiness), affective comfort in discussing overdose with peers and sharing information regarding overdose prevention resources (i.e., willingness), and reported possession and knowledge of how to use naloxone (i.e., ability). Readiness was measured via binary items asking participants to respond yes or no to the following statements: 1) “I have completed a training in how to use NARCAN (naloxone),” 2) “I know where to access NARCAN (naloxone) on my college/university campus,” and 3) “I currently have access to NARCAN (naloxone).” Willingness was measured via two 5-point Likert scale items ranging from 1 (strongly disagree) to 5 (strongly agree) asking participants to rate their level of agreement for the following statements: 1) “I would feel comfortable explaining to my peers where they can get NARCAN (naloxone)” and 2) “I would feel comfortable talking to my peers about overdose.” To capture ability, participants were asked to 1) respond yes or no to the following statement: “I know how to use NARCAN (naloxone)” and 2) rate their level of agreement with the following statement via Likert scale: “I would feel comfortable administering NARCAN (naloxone).”

Descriptive analyses were performed with survey responses to describe the demographic characteristics and levels of readiness, willingness, and ability to respond to opioid overdose. As all variables included binary and ordinal outcomes, descriptive statistics included frequencies and proportions indicating readiness, willingness, and ability for opioid overdose response preparedness. For Likert scale items, responses indicating level of agreement (i.e., “Agree” and “Strongly Agree”) were aggregated into single frequency and percentage outcomes. All quantitative analyses for these data were performed in R Studio.

#### 2.1.3 Survey participants

*N* = 352 students completed the cross-sectional survey. Most students surveyed were female (60.51%), Asian (49.72%), non-Hispanic/Latinx (73.01%), and on average were around 20 years old (*x* = 19.64, *sd* = 1.60). Most of our sample indicated that they were freshman (43.47%), followed by sophomores (25.28%), juniors (16.48%), seniors (11.36%), and graduate students (3.42%). Students predominantly indicated that they belonged to pre-professional and/or interest-based student organizations (34.66%), with a large proportion of students also indicating that they were not affiliated with a student organization (31.82%). See Table 1 for a breakdown of survey participant demographics.

**Table 1:** Survey demographics (*N* = 352).

Age in years (x, sd)	(19.64, 1.60)
Race (*n*, %)^*^	
American Indian/Alaskan Native	9 (2.56%)
Asian	175 (49.72%)
Native Hawaiian or Other Pacific Islander	2 (0.57%)
Black or African American	38 (10.80%)
White/Caucasian	123 (34.94)
Other	19 (5.40%)
Prefer not to answer	10 (2.84%)
Hispanic/Latinx (*n*, %)	
Yes	88 (25.00%)
No	257 (73.01%)
Prefer not to answer	7 (1.99%)
Gender (*n*, %)	
Male	123 (34.94%)
Female	213 (60.51%)
Non-binary	9 (2.56%)
Other	7 (1.98%)
Year of college (*n*, %)	
Freshman	153 (43.47%)
Sophomore	89 (25.28%)
Junior	58 (16.48%)
Senior	40 (11.36%)
Master's student	6 (1.71%)
Doctoral student	6 (1.71%)
Campus organization affiliation (*n*, %)^*^	
Not affiliated with an organization	112 (31.82%)
Greek life	27 (7.67%)
Spirit group	20 (5.68%)
Athletics	27 (7.67%)
Community organization	60 (17.05%)
Political or multicultural organization	31 (8.81%)
Art/event planning	11 (3.12%)
Pre-professional/interest group	122 (34.66%)
Other	45 (12.78%)

^*^–allowed to select more than one category.

x—mean.

sd—standard deviation.

*n*—frequency.

%—proportion of sample.

### 2.2 Focus groups

#### 2.2.1 Procedure

Focus group participants were recruited during tabling events and via social media, campus-based communications, flyers, and word-of-mouth. To capture perspectives from students with proximal experiences with drug overdose, participants needed to meet at least one of the following criteria (in addition to age, English fluency, and college enrollment criteria): (1) personally experienced an overdose, (2) personally witnessed an overdose, or (3) known someone close to them who experienced an overdose. Interested individuals completed a brief screening survey. Upon arriving at the focus group location, eligible participants were guided through informed consent and completed a demographic questionnaire. Each focus group lasted approximately 90 min. The purpose of these focus groups was to assess student perspectives on overdose prevention and response.

#### 2.2.2 Measures and analysis

The focus group facilitation guide was designed to gather students' thoughts and perceptions on overdose and overdose prevention resources at their university. Participants were introduced to the topic area, as well as definitions of particular drug classes (e.g., opioids, stimulants, hallucinogens) before beginning discussion. This discussion was broadly organized into questions regarding: (1) campus-based response to opioid overdose risk [e.g., What are your perspectives on (University)'s response to the overdose crisis?], (2) accessibility and effectiveness of campus-based resources for opioid overdose risk [e.g., How helpful are (University)'s resources for overdose prevention? Why?], (3) students' level of concern for opioid overdose [e.g., Did anyone hear that (county) declared a public health emergency due to overdose? What was your reaction to that?], and 4) solutions for improving student opioid overdose preparedness (e.g., How can resources be made more accessible? More helpful?). Audio recordings were transcribed verbatim; the transcripts were de-identified and cleaned. The process of cleaning included correcting spelling and grammatical errors in the transcription process, removing extraneous verbal musings (e.g., “um”, “er”, like”), correcting slang and informal language to bracketed full, formal terminology (e.g., “lotta” modified to “a lot of”), and including necessary contextual information in brackets to enhance readability and clarity of quotes.

Two trained research associates independently coded each transcript. Both research associates participated in follow-up meetings to compare codes and resolve coding discrepancies for each transcript. These finalized codes were used to build upon identified themes and sub-themes in NVivo 14. Second-line coding (i.e., summarizing subthemes within codes) was conducted by two authors, who are doctoral students with extensive training in qualitative methods (J.S., K.L.). We conducted an inductive-deductive thematic analysis of qualitative data and triangulated findings with survey data. Applied thematic analysis (24) and the RWA framework (9) guided the coding and interpretation process. The coding process revealed emergent themes that were categorized as facilitators, barriers, and solutions for improvement in each RWA domain related to overdose response preparedness.

#### 2.2.3 Focus group participants

*N* = 32 students with proximal experiences with drug overdose participated in a total of five focus groups. Most participants were female (62.50%), White (68.75%), Hispanic/Latinx (59.38%), and on average were around 20 years old (*x* = 20.40, *sd* = 2.11). Additionally, our sample was comprised of a mostly equal distribution of students with regard to year of college, with the slight majority being sophomores (28.12%). Lastly, most students reported belonging to pre-professional/special interest student organizations (53.12%). See Table 2 for a breakdown of focus group participant demographics.

**Table 2:** Focus group demographics (*N* = 32).

Age in years (x, sd)	(20.40, 2.11)
Race (*n*, %)^*^	
American Indian/Alaskan Native	1 (3.12%)
Asian	9 (28.13%)
Native Hawaiian or Other Pacific Islander	1 (3.12%)
Black or African American	3 (9.38%)
White/Caucasian	22 (68.75%)
Other	2 (6.25%)
Hispanic/Latinx (*n*, %)	
Yes	19 (59.38%)
No	13 (40.63%)
Gender (*n*, %)	
Male	9 (28.12%)
Female	20 (62.50%)
Non-binary	2 (6.25%)
Other	1 (3.12%)
Year of college (*n*, %)	
Freshman	8 (25.00%)
Sophomore	9 (28.12%)
Junior	7 (21.88%)
Senior	7 (21.88%)
Master's student	1 (3.12%)
Campus organization affiliation (*n*, %)^*^	
Not affiliated with an organization	8 (25.00%)
Greek life	0 (0.00%)
Spirit group	5 (15.62%)
Athletics	3 (9.38%)
Community organization	3 (9.38%)
Political or multicultural organization	8 (25.00%)
Art/event planning	3 (9.38%)
Pre-professional/interest group	17 (53.12%)

^*^–allowed to select more than one category.

x—mean.

sd—standard deviation.

*n*—frequency.

%—proportion of sample.

## 3 Results

### 3.1 Survey findings—low levels of readiness and ability, moderate levels of willingness

Cross-sectional survey findings highlighted low levels of preparedness for overdose response in readiness and ability, and moderate to high levels of willingness (Table 3). Less than a quarter of students knew where to access naloxone on campus (*n* = 83; 23.58%), only 14.20% (*n* = 50) indicated that they currently had access to naloxone, and only 10.51% (*n* = 37) indicated that they had received training in how to administer naloxone. Regarding willingness to respond to opioid overdose, most participants indicated that they would feel comfortable talking to their peers about overdose (*n* = 253; 71.88%) and explaining to their peers where naloxone could be accessed (*n* = 201; 57.11%). Last, ability for responding to opioid overdose was a deficit among students, with less than a quarter of participants indicating they knew how to use naloxone (*n* = 78; 23.01%) and less than a third reporting that they would feel comfortable administering naloxone (*n* = 110; 31.25%).

**Table 3:** Survey results—readiness, willingness, and ability in opioid overdose response preparedness (*N* = 352).

Readiness	
“I know where to access NARCAN (naloxone) on my college/university campus.” (*n*, %)	
No	267 (75.85%)
Yes	83 (23.58%)
Prefer not to respond	2 (0.57%)
“I currently have access to NARCAN (naloxone).” (*n*, %)	
No	300 (85.23%)
Yes	50 (14.20%)
Prefer not to respond	2 (0.57%)
“I have completed a training in how to use NARCAN (naloxone).” (*n*, %)	
No	313 (88.92%)
Yes	37 (10.51%)
Prefer not to respond	2 (0.57%)
Willingness	
“I would feel comfortable talking to my peers about overdose.” (*n*, %)	
Strongly disagree	8 (2.27%)
Disagree	42 (11.93%)
Neither agree nor disagree	49 (13.92%)
Agree	170 (48.30%)
Strongly agree	83 (23.58%)
“I would feel comfortable explaining to my peers where they can get NARCAN (naloxone).” (*n*, %)	
Strongly disagree	48 (13.64%)
Disagree	47 (13.35%)
Neither agree nor disagree	56 (15.91%)
Agree	121 (34.38%)
Strongly agree	80 (22.73%)
Ability	
“*I know how to use NARCAN (naloxone).”* (*n*, %)
No	271 (76.99%)
Yes	78 (22.16%)
Prefer not to respond	3 (0.85%)
“I would feel comfortable administering NARCAN.” (*n*, %)	
Strongly disagree	78 (22.16%)
Disagree	85 (24.15%)
Neither agree nor disagree	79 (22.44%)
Agree	73 (20.74%)
Strongly agree	37 (10.51%)

*n*—frequency.

%—proportion of sample.

### 3.2 Focus group findings—facilitating factors, challenges, and solutions for improvement

The following subsections describe emergent themes from applied thematic analysis of focus group in each of the domains of the RWA framework characterizing facilitating factors (i.e., factors that positively influenced preparedness for overdose response), challenging factors (i.e., factors that created barriers to preparedness for overdose response), and solutions for improving preparedness for overdose response (see Table 4 for additional supporting quotes). First, student perspectives characterizing readiness for overdose response preparedness are summarized. Second, student perspectives characterizing willingness for overdose response preparedness are summarized. Last, student perspectives characterizing ability for overdose response preparedness are summarized.

**Table 4:** Additional quotes describing emergent themes for readiness, willingness, and ability in overdose response preparedness among college students with proximal experiences to overdose (*N* = 32).

Domain	Emergent theme(s)	Representative quote(s)
Readiness for overdose response preparedness		
***Facilitators***	- Awareness of campus-based and non-campus-based overdose prevention and harm reduction resources, attending associated trainings	“*Not professionally, but the one that…I actually got because of [campus-based substance use resource]…there's a professor that teaches the [first and 2 year special interest course] here on campus...at the end of the semester, she always gives out Narcan to her students, so she gave it to us, too, which is pretty sick.” (Participant 2, Focus Group 3 [3,002])* “*I don't know who exactly put it [training] on, but it was at the [social work department]…I think that the training did a really good job. We learned a lot of the information about it.” [Participant 2, Focus Group 1 (1,002)]*
***Barriers***	- Gaps in awareness for campus-based overdose prevention resources by students due to a lack of advertising and dissemination of campus-based resources	“*…the only place I've ever heard of it [naloxone]…that's from…a student org telling me that, so it's not [university]…I haven't had personal experience with [university] being the ones, like, ‘This is where we offer it in case anyone needs it.' [Participant 6, Focus Group 2 (2,006)]* “*I'm an upperclassman…I haven't really heard too much about a university response to anything. I hear the news stories and things and that constantly, but as far as the university goes, not very much.” (Participant 2, Focus Group 3 [3,002])* “*I hear more about the mental health…I hear more about that than I do anything for…overdose.” [Participant 3, Focus Group 5 (5,003)]*.
***Solutions for improvement***	- Increase awareness of and access to naloxone distribution and overdose response training via various methods (e.g., increasing amount of naloxone distribution points, training opportunities via syllabi, orientations, targeting specific student populations, etc.) - Access to drug checking/testing resources	“*If it [naloxone] was at the student resource building…where people go to the doctor…because it would make sense…for…a drug overdose medication to be in the same facility where people go get…vaccines.” (Participant 4, Focus Group 4 [4,004])* “*… in a lot of our syllabi, they emphasize that there is a mental health facility. And most teachers emphasize, ‘If you need help, get it. Talk to D&A.' The disability and accommodations. I guess they could emphasize more, ‘There's also this [overdose response] resource if you need it.”' [Participant 2, Focus Group 5 (5,002)]* “*A lot more advertisement…If [university] sent emails or newsletters or made a notice about it. Because I get emails about [mental health resource]…They should just email it out. The students will see it.” [Participant 1, Focus Group 5 (5,001)]* “*…going to student orgs and making some sort of mandatory component…even if it's not just a purely a social org, but some sort of…a social component or threw a party on the weekends… ‘Look, here's Narcan. We're going to leave.0.0.50 doses here for you guys to use.'… ‘This is how you use it.”' [Participant 3, Focus Group 3 (3,003)]* “*I feel like what you said earlier about the [first-year course] discussions…that would be a good starting point because all freshmen are required to attend…they could use that time and that setting to go over resources.” [Participant 4, Focus Group 3 (3,004)]* “*All the freshmen, they have their orientation, so I don't know if that would be possible by adding another thing in their schedule for a demonstration or something like that, that they're forced to go to because it's part of their orientation schedule, then you can reach everyone and actually be in person where they're forced to listen” [Participant 1, Focus Group 4 (4,001)]* “*… make it required for Ras [resident advisors]. Because I know they do floor meetings and stuff… make it a requirement to talk about it… in the beginning of the year” [Participant 6, Focus Group 3 [3,006])*. “*The one thing...I think they should do is just provide testing kits and especially fent[anyl] [test] strips…they're cheap, easy to get, and…it's really saved a lot of people's lives.” [Participant 2, Focus Group 3 (3,002)]*. “*I think testing strips would be a great action of harm reduction.... I'm pretty sure [University] has a...substance abuse and recovery center...that place could have testing strips...any of the health services building could have testing strips...I think it would make sense to put them there.” [Participant 1, Focus Group 4 (4,001)]*
Willingness for overdose response preparedness		
***Facilitators***	- Personal investment in accessing resources for overdose response due to specific coursework, course tracks, or proximal experiences with overdose	“*I found out a lot from those connections...in social work… but also in the [early degree course]...I also interviewed [faculty] for a project, so…just those connections. Before that I didn't have a ton of information.” [Participant 2, Focus Group 1 (1,002)]*. “*I mean, I got lucky. I got a course that…went over…what addiction looks like. It's taught by a professor that's in long-term recovery, also. I just got lucky.” [Participant 5, Focus Group 3 (3,005)]* “*Only if it's [overdose] personally impacted them…I think that there's awareness if you have experiences [with overdose]” [Participant 1, Focus Group 4 (4,001)]*
***Barriers***	- Affective factors (e.g., stigma, fear, apathy) that influenced student's lack of awareness for overdose response resources and the overdose epidemic - Unwillingness by university to disseminate resources due to reputational and stigma-related concerns	“*They might be in with a group of people…who just don't use drugs ever, so they don't know how much it can affect a person and everyone around them.” [Participant 3, Focus Group 5 (5,003)]* “*…the ego of, like, ‘It's [overdose] not going to happen to me and then...if it does happen to me, I don't really care…” [Participant 1, Focus Group 2 [2,001])* “*…if there's a stigma around it [overdose] and everyone is uncomfortable talking about it, even if you have had experiences, or know someone, you might not speak up because of the stigma.” [Participant 4, Focus Group 4 (4,004)]* “*But there is that line that you have to be careful about. Just because obviously, you want people, if they're going to use drugs, to get it from a safer place, but you [university] can't be like, ‘Get it from a safe place.”' [Participant 1, Focus Group 5 (5,001)]*
***Solutions for improvement***	- Initiatives and programming for reducing individual and collective stigma related to overdose response preparedness (e.g., incorporating emotional appeal into resource dissemination, small group discussion/training formats)	“*I feel like emphasizing, ‘Would you know how to help a person?'…appeal to...their sense of humanity. ‘If you saw someone on the street, would you be able to help them?'... kind of...reaching, appealing to...their heart.” [Participant 3, Focus Group 5 (5,003)]* “*This [focus group]...a small discussion group would be useful, but maybe toward, people that are looking for the help specifically.” [Participant 5, Focus Group 3 (3,005)]*
Ability for overdose response preparedness		
***Facilitators***	-Granular knowledge for overdose response (e.g., information about potential naloxone toxicity)	“*The biggest thing that I actually learned recently…is that you can put a lot of Narcan in somebody before it actually hurts them—so if anyone's unconscious and that's a thing you're worried about, you can just give them Narcan.” [Participant 1, Focus Group 2 (2,001)]*
***Barriers***	-Insufficient prior experiences with overdose response trainings and resources	“*Yeah, it's [training received] better than nothing. I don't know that I would be able to implement it in real life.” [Participant 4, Focus Group 4 (4004)]* “*You need to have some prior experience and some handling with the tools for you [to] actually be able to use it good...you really won't know that from the instructions that you're reading on the label in an emergency.” [Participant 8, Focus Group 3 (3,008)]* “*...my friends basically they didn't want to do those trainings [orientation substance use modules] and just skipped through it, so they missed out on some of the information that was provided…I think a lot of students don't take that stuff seriously.” [Participant 2, Focus Group 1 (1,002)]*
***Solutions for improvement***	-Improving level of detail and delivery modalities in overdose response trainings (e.g. peer-to-peer trainings)	“*If they [orientation substance use modules]...talked about exactly how to administer Narcan and what to look for, I think it would bring a lot more realism to the issue and perhaps prevent the...problem of freshmen just kind of skipping through it” [Participant 9, Focus Group 3 (3,009)]* “*....it could also be useful if you had maybe students who were previously trained to teach how to use it [naloxone]. If there was more of a closer element...And you would just go over...how to use it and what to expect.” [Participant 4, Focus Group 5 (5,004)]*

#### 3.2.1 Readiness for overdose response preparedness

##### 3.2.1.1 Facilitating factors

Emergent themes from focus groups highlighted how readiness for responding to opioid overdose was facilitated by awareness of campus-based overdose prevention and harm reduction resources, as well as attending associated trainings at campus-based resources. One participant noted positive perceptions about naloxone distribution at the campus library: “The Narcan at [campus-based library] has definitely been…a big step that I did not think [University] was going to take…I think that has been a really good part” (Participant 1, Focus Group 2 [2001]). Another participant noted becoming aware of campus-based naloxone distribution and availability when they moved into the residential dorms on campus:

“I got that information when I first moved in [to dorms]…‘If you ever experience...anybody with overdose in the dorms, we [dorm staff] are equipped with Narcan.' And that was the first time I ever heard of it.” (Participant 3, Focus Group 2 [2003])

In addition, several participants indicated that they were aware of and had taken part in opportunities to receive training on how to administer naloxone and protocols for opioid overdose response. Several participants noted that they received training in naloxone administration and opioid overdose response from campus-based entities that specialized in providing resources for student substance use:

“I went to a training…I think it was [campus-based student recovery service]…I went to their building and I received two Narcans…I went through the training and I know how to use it.” (Participant 1, Focus Group 1 [1001])

Other students noted that there were departments on campus (i.e., pharmacy) that hosted trainings to improve readiness for naloxone administration:

“The [pharmacy department] has a program that is doing Narcan trainings…. They came in and showed us how to administer Narcan.” (Participant 10, Focus Group 3 [3010])

Focus group participants also reported accessing naloxone and overdose response trainings that were offered by non-campus-based entities, including community-based harm reduction organizations:

“I took a training with [community-based harm reduction organization] virtually and they showed how to do both the nasal and the shot one.” (Participant 4, Focus Group 1 [1004]).

##### 3.2.1.2 Barriers

Emergent themes describing barriers or factors that challenged readiness to respond to an opioid overdose characterized a lack of awareness for campus-based overdose prevention resources by students, which was primarily reported to be due to a lack of advertising and dissemination of campus-based resources for opioid overdose response. One participant bluntly stated, “I think it's pretty useful to have Narcan but if no one knows about it, then it's not useful” (Focus Group 4, Participant 2 [4002]). Other participants noted that awareness and readiness barriers were related to their university not advertising or promoting awareness about associated resources:

“I agree that it's not widely publicized that [university] is doing anything. I didn't know that about the Narcan being available at the [campus-based library]. I also think that further harm reduction education would be really beneficial.” (Participant 4, Focus Group 4 [4004]).

Focus group participants also clarified that gaps in awareness and subsequent readiness for overdose response were experienced by students regardless of how far they were into their degree coursework:

“I'm a freshman and I haven't even heard stuff from older students, which you'd think you would. But I think they don't even know either. So, I think just over the past three to 4 years most of us just don't know anything… about what [University] has to offer for it [overdose prevention resources].” (Participant 1, Focus Group 5 [5001])

Several students shared that although they noted awareness and dissemination of other resources led by their university, they did not see similar efforts to disseminate overdose prevention resources. One participant described the following about their orientation process and a lack of a focus on substance use resources:

“They [university] did mention a lot about mental health resources…which is obviously good. But I don't remember anything about drug use.” (Participant 1, Focus Group 5 [5001])

##### 3.2.1.3 Solutions for Improvement

Emergent themes also highlighted student perceptions regarding solutions for improving readiness for overdose prevention and response. Namely, students reported increasing awareness of and access to naloxone distribution and overdose response training via various methods. A commonly reported solution for improving community-level readiness was increasing the availability of naloxone on campus. One participant reported that naloxone should be made as available as other emergency response tools, such as automated external defibrillators, in campus buildings:

“…in every building, we have an AED [automated external defibrillator] and a bleeding control kit. So why can we not have a Narcan kit also in the exact same spot?” (Participant 10, Focus Group 3 [3010])

Several participants offered specific locations frequented by large amounts of students (e.g., recreational sports centers) that could be targeted specifically for expanding campus-based naloxone distribution and other resources for overdose response preparedness:

“I think better money could be spent to providing the resources. Not only in the [campus-based library], but also in [healthcare clinic]. Also, in [campus-based recreational sports center].” [Participant 3, Focus Group 5 (3005)]

Another solution endorsed by focus group participants described several mechanisms that their university could leverage to increase awareness and collective readiness for overdose response. Several participants noted that resources are often discussed on the 1 day of classes within a given semester and that faculty could incorporate information about campus-based overdose response resources:

“…it's as simple as telling professors, ‘On your syllabus, say, ‘Hey, explain where the resources are.” It takes 5 min. You could explain to someone where to get Narcan or…explain what an overdose looks like.” [Participant 10, Focus Group 3 (3010)]

Several students also noted that mass communication methods for spreading awareness of overdose response resources are viable methods for increasing collective readiness:

“…just send out a mass email…, ‘These are what the resources are, these are what the resources can do for you if you or someone else you know might be at risk of overdosing.'…there are plenty of hotlines…that you could call that you could easily send out that would cost the university no money and would quickly spread the word about…resources to the entire student population.” [Participant 1, Focus Group 4 (4001)]

Students also underscored the importance of targeting readiness for opioid overdose response among student subpopulations that might have heightened substance use and associated overdose risk (e.g., younger, incoming students; Greek life and spirit groups). Several participants noted targeting student organizations with social or party-specific activities for scaling up resources for overdose response preparedness:

“I was talking about… targeting… organized party groups essentially…if overdosing happens in social party settings, then going to these groups that are organized…have them sit through a lecture… They're registered in the [university] database of orgs…they can be found, reached, and given stuff [overdose response resources].” [Participant 9, Focus Group 3 (3009]))

Students also stressed that providing awareness and training for overdose response in settings that intersect with incoming student populations could help improve collective readiness and ameliorate barriers related to a lack of institutional dissemination. Participants noted that the possibility of infusing overdose response trainings into 1 year courses and orientation would facilitate improving student readiness for overdose response:

“I think it would be really beneficial….to require… drug overdose…orientation modules…you had to do it within the first semester you were here on campus, and I think that would be really useful to implement something like that.” [Participant 1, Focus Group 4 (4001)]

Participants noted that ensuring that resident advisors (RAs) were trained in naloxone administration would improve collective readiness for opioid overdose response among younger students living in dorms on campus:

“I feel like… one thing could also just be… making all RAs on the campus—giving them training in how to administer Narcan….because then you have Narcan in every venue. You have a RA in every…hallway. And that can help.” [Participant 7, Focus Group 3 (3007)]

Finally, students indicated that they would benefit from campus-based access to drug checking and testing strategies to check for adulterants to mitigate overdose risk, which would improve community-level readiness for effective overdose response preparedness:

“I agree with him. I have not heard about the university's specific response, but one thing I know they don't do and should do is offer [drug] testing. Because they're unable to stop students from using drugs, especially ones that could be laced with opioids, but...it could definitely reduce the amount of casualties.” [Participant 3, Focus Group 3 (3003)]

#### 3.2.2 Willingness for overdose response preparedness

##### 3.2.2.1 Facilitating factors

Emergent themes characterizing facilitating factors for willingness in responding to an opioid overdose centered around the phenomenon that certain subgroups of students had a greater level of personal investment in accessing resources for overdose response. Several students noted that their interest in coursework in health-related tracks and personal interest in contributing to substance use education facilitated opportunities to receive education and awareness about overdose response:

“I would feel comfortable talking about it in my specific courses. I'm a bio-med major, and sometimes our class will talk about hard things like ethical decisions, or overdose, for example. I would feel comfortable talking about it [overdose] there” [Participant 2, Focus Group 5 (5002)]

In addition, several participants noted that their proximal experiences with overdose (i.e., personally experiencing, witnessing, or knowing someone who had experienced overdose) bolstered willingness to receive more awareness and preparedness for overdose response. One participant shared that shared that they personally sought to become more aware of the overdose crisis and associated resources after he had a personal experience related to overdose:

“They [students] seem to realize it's a problem, but it's never happened to them personally…the main reason I started taking a step further was because I had a personal experience with it.” [Participant 7, Focus Group 3 (3007)].

##### 3.2.2.2 Barriers

Emergent themes characterizing barriers and challenges to student willingness for preparedness in overdose response detailed affective factors that influenced student's lack of awareness for overdose response resources and the overdose epidemic, as well as perceptions that the university was unwilling to disseminate resources due to reputational and stigma-related concerns. Several participants shared that among students who did not use substances, there was a collective deprioritization of overdose response preparedness due to not participating in drug use or party culture:

“I think there's a great amount of people who know, but don't care because they don't use, or maybe they think that people around them don't either...So I feel like they think, ‘Oh. Because I'm never going to use it, it will be fine.”' [Participant 1, Focus Group 5 (5001)]

Another participant highlighted the phenomenon of students not engaged in substance use as apathetic toward overdose response preparedness due to not having a personal or proximal experience with drug overdose:

“Most people I know don't have that personal experience, and when they think about the opioid crisis and overdose issues, the extent of their thinking is, ‘I'm going to make choices for myself that, uh, you know, kind of save me or… remove me from this danger,' and that's it. So, they make those choices, and they don't think twice about, other people they know who don't make that choice or how they could help other people that are suffering from this problem.” [Participant 3, Focus Group 3 (3003)]

Several participants also noted that there was deprioritization for overdose response preparedness among students that were using drugs or participating in party culture, which was characterized by feelings of impulsivity and invincibility to the risks of overdose:

“[Overdose is] a lot more common than we think. And no one would know what to do just because…they don't think it's going to happen to them….they think they know what they're doing, but they don't realize how hard it is to not know what you're doing.” (Participant 2, Focus Group 5 (5002)].

One student shared deprioritizing overdose prevention measures (e.g., testing drugs) leads to instances of student substance use where they are unaware of the content of the drugs they are taking:

“‘Oh, well, why would I want to test my drugs?'...it's readily available to me right now here at a party, so I'm just going to do it and not really think much about it because if everyone around you is also participating, you just...also want to participate.” [Participant 5, Focus Group 2 (2005)]

Students also highlighted concerns for other students struggling with substance use but who experienced fear and stigma-related barriers to accessing campus-based resources for overdose response:

“From my experience...students at [university] who do opiates and stuff, they'll take the Narcan but they're not going to actively get help from the school…because there's a bit of a stigma, and they're scared of being found out.” [Participant 4, Focus Group 5 (5004)]

Another participant clarified that the stigma associated with drug use has a challenging effect on willingness for overdose response preparedness was amplified due to concerns that academic standing would be threatened:

“You don't want to be… [a] nasty person who uses drugs in college....no one wants their professors to know they're using drugs…you have…social and academic stigma…‘If I get caught, I might lose my degree.”' [Participant 6, Focus Group 3 (3006)]

Focus group participants also noted perceptions that indicated that the university did not see community-level overdose response as a priority due to the social risk inherent in endorsing and disseminating harm reduction and safe use resources. One participant offered the following when considering university promotion of overdose response and drug checking resources:

“Is [University] going to be like, ‘Come test your drugs, you know, make sure they're safe.' Probably not….it probably wouldn't look good for the university.” [Participant 3, Focus Group 4 (4003)]

##### 3.2.2.3 Solutions for improvement

Solutions offered by focus group participants to mitigate challenges related to willingness centered on initiatives and programming for reducing individual and collective stigma related to overdose response preparedness. Students discussed the importance of messaging and overdose response trainings that appealed to affective and emotional prioritization for increasing willingness among students:

“…if people really sat down and talked about it and their experiences…people will care. It's the human condition of empathy and compassion. We should hone in more on that sense of connecting with people vs. shoving information down your throat. Because if you talk about these experiences, people would want to know.” [Participant 1, Focus Group 1 (1001)]

Similarly, students discussed the importance of offering community conversations and opportunities for bolstering overdose response preparedness in formats that were casual and in small group formats to facilitate affective comfort accessing resources:

“I think that it being more casual kind of makes it easier for people because if it's a really serious overdose then people react more strongly to it and I feel like they'd just be more open to talking about it if it was more casual.” [Participant 3, Focus Group 4 (4003)].

One student also noted the need for stigma-reducing initiatives carried out by the university in order to mitigate fear and stigma-related barriers to accessing campus-based overdose response resources:

“I think de-stigmatizing drugs is really important because people are unwilling to receive help for something that they feel that they'll be shamed or criminalized for doing. So obviously if there was a larger movement to address the way that substances are perceived in [University] or just in general, that could help in terms of increasing accessibility.” [Participant 1, Focus Group 4 (4001)]

#### 3.2.3 Ability for overdose response preparedness

##### 3.2.3.1 Facilitating factors

Emergent themes characterizing facilitating factors bolstering ability to respond to overdose included protocol-specific knowledge expressed by students who had attended overdose response trainings. Several participants shared that they had obtained granular information about naloxone and overdose symptomatology from trainings attended to become equipped in overdose response preparedness. One

participant noted that they learned about the signs and symptomatology of an opioid overdose and that this improved their confidence in overdose response preparedness:

“I think mine was pretty good because it talked about how to identify if an overdose is actually happening.... how it could look if it's super dramatized. But in reality it could just look like someone's asleep….or it could be... blue in the lips or something like that. So...it talked about how to identify them, which I was missing. And so that was good from the training.” [Participant 1, Focus Group 1 (1001)]

##### 3.2.3.2 Barriers

Emergent themes characterizing barriers and challenging factors to ability for opioid overdose response preparedness were defined by perceptions that previous overdose response trainings were insufficient for preparing students to respond if they actually encountered an overdose:

“…mine [overdose response training] wasn't very thorough. It was just kind of simple direction… I guess I wouldn't really consider mine training.” [Participant 3, Focus Group 2 (2003)]

Several students also underscored the limitations of written materials describing the steps in overdose response as insufficient in boosting confidence and ability to implement overdose response steps:

“just the written steps aren't enough. I think that actually practicing it...‘What does this drill look like? How do you recognize it on someone that's mirroring those symptoms, those sorts of things.”' [Participant 1, Focus Group 4 (4001)]

Students underscored how substance use orientation modules that were offered by the university were largely ineffective in strengthening perceptions of their ability in overdose response preparedness. In discussing their orientation substance use education modules, one participant shared, “there was nothing about drug overdose stuff that I remember.” [Participant 2, Focus Group 2 (2002)]. Other participants noted that the current substance use orientation modules were seen as a hindrance to students, which limited their engagement in the material:

“I think it's something [orientation substance use modules] that students see as a hassle. And it just creates more frustration, so they're less likely to, 1. Listen, 2. Even care about it…because it's something that they have seen as an annoyance…And I just don't think it's very helpful.” [Participant 4, Focus Group 1 (1004)]

##### 3.2.3.3 Solutions for improvement

Solutions offered by students to enhance ability for overdose response preparedness centered on improving the level of detail and delivery modalities in overdose response trainings. Several participants underscored specific knowledge in overdose response protocol that they would need to feel confident in their ability in overdose response, such as symptoms, demonstrating administering naloxone, and other necessary information (e.g., sternal rub):

“I think the first thing is...knowing what exactly the symptoms are…it would be hard to know the difference [between] [if] someone‘s...just nodding off—or falling asleep, or if they're overdosing…and the signs of that...and then obviously how to use the Narcan…And he [another participant] was talking about a sternum rub. I have no idea what that is….So, I'd have to learn every step in detail to know exactly...how to tell someone [is] overdosing and then how to deal with it” [Participant 2, Focus Group 4 (2004)]

Another participant noted that learning about dosages of particular substances that increase risk of experiencing overdose would be helpful information to include in overdose response trainings:

“And they [trainers] show you, what the actual, dosage—or...amount a person can have before they have severe effects...to be able to identify, harmful drugs because...I know what's going on in [City] right now is the fentanyl crisis…a lot of people don't know that they take a random drug at a party and they don't know that it's fentanyl.“ [Participant 2, Focus Group 5 (5002)]

Several students highlighted the importance of involving peer-to-peer training opportunities. Multiple participants noted the possibility of having trained student peers to be able to train other students on how to administer naloxone:

“…anybody who knows exactly how to use it [naloxone] can teach someone…So just someone with credibility…. I think even peer-to-peer education would be sufficient because it's a simple enough process in theory as long as the training...suits the learner… But if I had a peer that went through the steps with me, that would be more than enough.” [Participant 1, Focus Group 4 (4001)]

One participant even mentioned that they would be willing to serve as a peer educator for naloxone administration and would offer trainings to peers in student organizations:

“I would have no problem…if I had proper training and I knew the proper resources I would go to my friend who is an officer in [Student Organization] and tell her, ‘Hey, you should really administer this [naloxone training]' and I'm sure, as she works as an officer, she would.” [Participant 2, Focus Group 5 (5002)]

One student also clarified that trainings that are led by individuals with expertise in overdose response would enhance the effectiveness of trainings and potential for implementing actionable steps to respond to an overdose situation:

“But if you do it in...a smaller environment, where...the people who are presenting have experiences...have some sort of experience [with overdose], and obviously are educated enough on the topic to....give some sort of education, and then also...show how to administer Narcan, I think...that would be very helpful.” [Participant 5, Focus Group 1 (1005)]

## 4 Discussion

Comprehensive findings from survey and focus groups align with existing research documenting low levels of readiness and ability among college students to respond to opioid overdose (14, 15). Cross-sectional survey findings highlighted low levels of awareness for campus-based naloxone distribution and associated readiness for opioid overdose response, as well as low levels of knowledge and confidence in ability to administer naloxone. Conversely, surveyed students indicated moderate-high levels of willingness to discuss overdose with peers and share information regarding where to access naloxone. Focus groups with students with proximal overdose experiences highlighted gaps in dissemination of resources and trainings to bolster readiness for opioid overdose, individual and collective stigma toward overdose response preparedness among students that impacted willingness to respond to overdose, and low levels of skill and confidence to be able to respond to overdose events due to ineffective training. Students indicated that university education initiatives and resources could benefit from broader dissemination, inclusion of other drug-related content, and leadership from peers trained in overdose response and other lived expertise. In particular, students offered a multitude of solutions to improve on the low levels of collective readiness for opioid overdose response preparedness in increasing availability of naloxone and targeting existing university infrastructure to disseminate education regarding overdose response preparedness (i.e., orientation).

Suggested solutions for improving readiness for overdose response preparedness are supported by policies implemented in U.S. states and design considerations for increasing awareness for overdose response resources on college campuses. Existing resources familiar to focus group participants were consistent with those typically provided by universities, including naloxone distribution points and a mandated module upon matriculation which discusses problematic alcohol use (25); however, students reported that matriculation modules did not discuss drug overdose and that university-led dissemination efforts to increase readiness for overdose response preparedness were limited. In contrast, statewide policies mandating that public universities stock naloxone on campus and training for incoming students show promise in increasing baseline levels of readiness (26, 27). Policies to expand access to drug testing and checking resources (i.e., fentanyl testing strips) among college students attending state universities (28, 29) reflect solutions suggested by study participants in expanding access to drug checking resources in overdose response preparedness. In addition, efforts to design campus-based naloxone distribution models highlight the importance of centralizing campus-wide communications and providing standardized avenues for accessing education on overdose response (13). Universities should utilize existing centralized communication mechanisms like mass email and course syllabi to combat stigma and raise awareness, ultimately increasing readiness and willingness among students (13). Storing naloxone alongside other emergency preparedness tools such as automated external defibrillators may be a feasible, impactful, and intuitive strategy to increase campus readiness for overdose response (30–32). Efforts to improve readiness for overdose response also should be mindful that distribution and access to naloxone be paired with efforts to expand awareness of overdose education resources, and that these should not be treated as separate initiatives (33).

In addition, students supported efforts to improve affective and cognitive prioritization of overdose response preparedness among students to mitigate stigma-related barriers. Prior work underscores the importance of stigma-free spaces to access overdose response resources (31). Stigma-related to opioid use disorder among student populations is characterized by thoughts that dispensing naloxone encourages drug use and unwillingness to work or live with people who use drugs (31, 34). However, studies also note lower levels of stigmatizing beliefs among students who support campus-based naloxone distribution (31). Although survey findings reflected high levels of willingness to discuss overdose and related resources, focus group participants indicated individual and collective stigma impacting willingness in increasing preparedness for overdose response. Focus group participants discussed hesitation to talk to other students about drug use and overdose prevention out of fear of judgment, a finding consistent with other explorations of barriers to naloxone training on college campuses (13). Students also expressed perceptions reflective of other students on campus that overdose was not personally relevant to them and thus, there is a lack of peer conversations about overdose. Students suggested that messaging that leverages empathy and humanism in resource dissemination and delivery of specific trainings for overdose response preparedness could bolster willingness in relation to overdose response preparedness. Suggestions to infuse affective and emotional messaging about opioid overdose preparedness reflect that presenting threat-only messages create fear and that empowering content can increase motivation to engage in overdose prevention measures (35). However, stigma-related barriers to accessing overdose response resources for students experiencing substance use challenges persist (36), highlighting the need for community-level efforts to reduce stigma associated with drug use and help-seeking. Study participants also indicated the need to improve trainings for overdose response preparedness, particularly through including peer champions with lived expertise who could provide granular, specific knowledge related to improving overdose response abilities.

Multiple studies have supported the effectiveness of peer-led education and training (37, 38) to increase overdose response preparedness on university and college campuses. Focus group participants attributed their limited ability to training formats that were not engaging and lack of real-world examples in training modules. Existing research has found that integrating interactive components such as role-play, simulation, and discussion into overdose response training may increase college students' skill development and confidence in responding to overdose (30). In addition to reducing stigma, college students have reported that peer-led training has provided the knowledge needed to effectively respond to overdose (38). Incorporating lived experience has shown to be beneficial in reaching those at highest risk and conveying detailed practical knowledge for overdose response preparedness (e.g., overdose symptomatology, what to expect if calling emergency medical services) (39). Following suggestions from focus group participants, the expertise of students in health sciences course tracks and students with proximal experiences to drug overdose in providing peer-to-peer training may facilitate increasing students' ability to respond to overdose.

### 4.1 Limitations

This study has several limitations. First, we used convenience sampling for the survey which may influence the generalizability of these findings. Future research should examine readiness, willingness, and ability to respond to overdose using more rigorous sampling methods. Second, this study was conducted at a single college campus and findings may not be generalizable to college campuses of different size, student demographics, and/or available resources for overdose response preparedness. Third, the nature of data collection via focus groups may have limited some participants' willingness to disclose perspectives relevant to stigma and overdose. Last, the perspectives of our focus groups are limited in their ability to speak to barriers to awareness experienced by students without proximal experiences to overdose. However, we believe that the perspectives offered by our participants provide necessary insight and points of consideration for any overdose prevention programming led by universities.

## 5 Conclusions

Study findings highlight an urgent need for universities to tailor efforts to improve readiness, willingness, and ability to respond to overdose. Universities should support readiness, willingness, and ability to respond to overdose at the individual and structural levels through increased access to naloxone across campus, greater advertising of resources through centralized communication mechanisms, peer-to-peer education, and interactive overdose response training formats. Efforts to improve overdose response preparedness should also draw on the knowledge of students with previous experience with overdose.

## Data Availability

The datasets presented in this article are not readily available because of the sensitive nature of discussion topics and the student population that was engaged with for focus groups. Requests to access the datasets should be directed to Jake Samora; jake.samora@austin.utexas.edu.
